# Ganglioside Synthesis by Plasma Membrane-Associated Sialyltransferase in Macrophages

**DOI:** 10.3390/ijms21031063

**Published:** 2020-02-05

**Authors:** Aldo A. Vilcaes, Eduardo Garbarino-Pico, Vanina Torres Demichelis, Jose L. Daniotti

**Affiliations:** 1 Centro de Investigaciones en Química Biológica de Córdoba (CIQUIBIC), CONICET. Universidad Nacional de Córdoba, Córdoba X5000HUA, Argentina; garba@fcq.unc.edu.ar (E.G.-P.); vtorres@fcq.unc.edu.ar (V.T.D.); 2 Departamento de Química Biológica Ranwel Caputto, Facultad de Ciencias Químicas, Universidad Nacional de Córdoba, Córdoba X5000HUA, Argentina

**Keywords:** gangliosides, sialyltransferase, macrophage, cis-catalytic activity, nitric oxide, glycosphingolipids

## Abstract

Gangliosides are constituents of the mammalian cell membranes and participate in the inflammatory response. However, little is known about the presence and enzymatic activity of ganglioside sialyltransferases at the cell surface of macrophages, one of the most important immune cells involved in the innate inflammatory process. In the present study, using biochemical and fluorescent microscopy approaches, we found that endogenous ST8Sia-I is present at the plasma membrane (ecto-ST8Sia-I) of murine macrophage RAW264.7 cells. Moreover, ecto-ST8Sia-I can synthetize GD3 ganglioside at the cell surface in lipopolysaccharide (LPS)-stimulated macrophages even when LPS-stimulated macrophages reduced the total ST8Sia-I expression levels. Besides, cotreatment of LPS with an inhibitor of nitric oxide (NO) synthase recovered the ecto-ST8Sia-I expression, suggesting that NO production is involved in the reduction of ST8Sia-I expression. The diminution of ST8Sia-I expression in LPS-stimulated macrophages correlated with a reduction of GD3 and GM1 gangliosides and with an increment of GD1a. Taken together, the data supports the presence and activity of sialyltransferases at the plasma membrane of RAW264.7 cells. The variations of ecto-ST8Sia-I and ganglioside levels in stimulated macrophages constitutes a promissory pathway to further explore the physiological role of this and others ganglioside metabolism-related enzymes at the cell surface during the immune response.

## 1. Introduction

Gangliosides are sialic acid-containing glycosphingolipids that are constituents of mammalian cell membranes. They are mainly located in the outer layer of the plasma membrane of vertebrate cells [1,2]. These sialosylated molecules are involved in many physiological processes, including growth, differentiation, migration and apoptosis through modulation of both cell signalling processes and cell-to-cell and cell-to-matrix interactions [3,4,5,6]. A recent report provides evidence of the role of GD3 synthase (ST8Sia-I) in bone metabolism in animals and suggests that altered ganglioside metabolism is associated with this bone loss during aging [7]. Alterations of ganglioside composition have been shown to occur upon transformation and upon cellular differentiation [8]. In addition, in melanoma cells, specific gangliosides are associated with the adapter molecules of the cell surface promoting their activation during adhesion [9,10]. In particular, the immunological and structural attributes of gangliosides from macrophages are of particular importance and have been the subject of several studies [11]. Recent findings suggest that tumor-shed ganglioside is a secretory factor regulating the phenotype of macrophages and consequently enhancing angiogenesis [12]. In addition, exogenous GM3 ganglioside can suppress the lipopolysaccharide (LPS)-induced inflammatory response in RAW264.7 cell line [13]. In macrophages and microglia, the ganglioside content available in the extracellular space modulates Toll-like receptor 4 (TLR4) mediated stimulation [14,15]. Existing evidence supporting the role of gangliosides as modulators of immune cells activation has been obtained by adding exogenous gangliosides to cultured cells. Very little is known about the putative changes in the expression and localization pattern of endogenous gangliosides after macrophage activation [16,17]. Furthermore, the enzymatic changes underlying ganglioside modification remain largely unknown.

It is very well documented that ganglioside pattern of expression is mainly determined by glycolipid glycosyltransferases regulation at the transcriptional and post-transcriptional levels and by specific transport proteins in the lumen of the Golgi complex [1,18,19,20,21]. In recent years, the advances in biochemical and molecular biology techniques have produced a notable growth in our knowledge about the presence and significance of ganglioside glycosyltransferases and glycohydrolases associated with the outer layer of the plasma membrane. In this respect, our laboratory demonstrated the expression of ST8Sia-I at the cell surface of epithelial (ectopic ST8Sia-I expression) and melanoma (endogenous ST8Sia-I expression) cell lines [22]. This membrane-integrated sialyltransferase was able to synthetize GD3 at the cell surface (cis-catalytic activity). Later, it was also demonstrated that ecto-ST8Sia-I is able to sialylate immobilized GM3 onto a solid surface or GM3 belonging to the surface of neighboring cells (trans-catalytic activity) [23,24].

The sialylation level affects the activity of immune cells under different physiological and pathological conditions. In macrophages, the complex process of inflammation after LPS stimulation is associated with changes in sialic acids content at the plasma membrane [25,26]. In this sense, the sialyltransferases ST3Gal-I and ST6Gal-I [27] are upregulated and downregulated during LPS macrophage activation, respectively. In addition, a new role for sialidase NEU1 on the regulation of TLR4 was demonstrated [28]. However, there is little information about the presence of endogenous ecto-ST8-SiaI in macrophages [7].

In the present work, we extend our previous findings by elucidating the presence and activity of ecto-ST8Sia-I at the plasma membrane of unstimulated and LPS-stimulated macrophages. The results show that endogenous ST8Sia-I, localized at the cell surface of RAW264.7 macrophages, was able to transfer a sialic acid residue onto GM3 to synthesize GD3 (cis-catalytic activity). Stimulation with LPS led to a reduction on ST8Sia-I levels without affecting its cis-catalytic activity. Addition of an inhibitor of the nitric oxide (NO) synthase reverted the LPS effects on ST8Sia-I levels, suggesting that NO formation is involved in the expression changes of this enzyme in response to LPS. The proinflammatory stimulation of macrophages with LPS also reduced the expression endogenous GD3 and GM1 gangliosides and increased GM3 and GD1a gangliosides. Taken together, our findings demonstrate the presence and activity of ST8Sia-I at the plasma membrane of RAW264.7 macrophages. The cell surface levels of gangliosides and associated enzymes, thus, emerges as a new and promissory pathway involved in the activation of macrophages. The exact physiological role and impact of this pathway in the immune response will be the subject of further research.

## 2. Results

### 2.1. Endogenous ST8Sia-I Subcellular Localization in Macrophages

**Figure 1:**
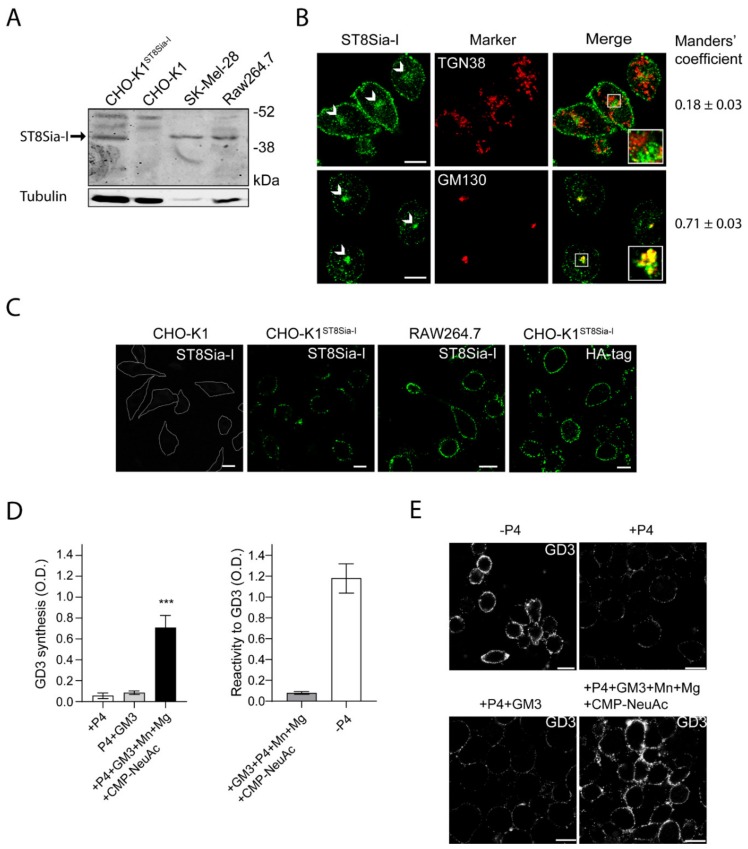
Expression and subcellular localization of ST8Sia-I. (**A**) Western blot of total cell homogenate from CHO-K1, CHO-K1^ST8Sia-I^, SK-MEL-28 and RAW264.7 cells. Detection of ST8Sia-I was carried out using anti-ST8Sia-I polyclonal antibody. Tubulin was used as a loading control. Data are representative for three independent experiments. (**B**) RAW264.7 cells were fixed and permeabilized before immunostaining with antibodies to ST8Sia-I, GM130 (cis-Golgi Marker) and TGN38 (trans-Golgi network marker). The Golgi structure is indicated (arrows). Insets in merge panels show details at a higher magnification. Manders’ coefficient (red overlapped with green) is indicated at the bottom of each image. (**C**) CHO-K1, CHO-K1^ST8Sia-I^ and RAW264.7 cells were immunostained with antibody to ST8Sia-I and visualized by confocal microscopy. Alternatively, CHO-K1^ST8Sia-I^ cells were immunostained with an antibody to HA (fourth panel). The boundaries of the CHO-K1 cells are shown with white lines. (**D**) Detection of GD3 synthesis at the cell surface of RAW264.7 cells by ELISA. RAW264.7 cells were treated with P4 for 4 days (+P4). Then, cells were incubated with 25 µM GM3, washed, and incubated at 37 °C for 60 min in a medium containing only Dulbecco’s Modified Eagle Medium (DMEN) (+P4+GM3) or in a medium containing CMP-NeuAc, Mn^+2^ and Mg^+2^ (left bar graph). The P4 inhibitor remained present throughout the experiments. Cells were washed and incubated with TruStain fcX for 10 min on ice and then incubated with antibody to GD3 (R24) at 4 °C for 60 min, fixed and incubated with secondary antibody conjugated to Horseradish peroxidase (HRP) at 4 °C. After washes, HRP activity was revealed and the optic density measured at 490 nm as indicated in the Materials and methods section. In right bar graph, as a positive control of the assay, RAW264.7 cells were grown on 96-well polystyrene flat-bottom plates without P4 (-P4) for 4 days and GD3 expression was revealed by Enzyme-linked immunosorbent assay. As a negative control of the assay GM3 (0.05 nmol) was coated on polystyrene microtitration plates in methanol and dried at 37 °C. After ganglioside coating, wells were saturated with 2% Bovine serum albumin (BSA) in Phosphate-buffered saline (PBS), washed thoroughly with PBS and GD3 immunodetected by ELISA. (**E**) RAW264.7 cells treated with P4 or without P4 (-P4, first panel) for 4 days were incubated with 25 µM GM3, washed, and incubated at 37 °C for 60 min in a medium containing only DMEM (+P4+GM3) or containing CMP-NeuAc, Mn^+2^ and Mg^+2^ (+P4+GM3+Mn+Mg+CMP-NeuAc). The P4 inhibitor remained present throughout the experiments. Then, cells were washed, immunostained with antibody to GD3, fixed and incubated with secondary antibody conjugated to Alexa488. Representative confocal sections of 0.8 μm taken parallel to the coverslip are shown. The fluorescence micrographs are representative of three independent experiments. Data are expressed as mean ± S.E.M. from three independent experiments, each in triplicate. Scale bar: 10 µm.

Endogenous expression levels of ecto-ST8Sia-I was detected by Western blot in murine RAW264.7 macrophages. Previous studies have shown that the level of ST8Sia-I mRNA is distinctively high in human melanoma cell lines, such as SK-MEL-28 [29]. In agreement, our Western blot analysis showed a clear band of 44–47 kDa in SK-Mel-28 cell line, indicating endogenous expression of ST8Sia-I (Figure 1A). As a control, the CHO-K1 cell line which lacks ST8Sia-I was used (Figure 1A). Specific antibody labeling was confirmed by cell transfection with a Human influenza hemagglutinin (HA)-tagged version of the enzyme (CHO-K1^ST8Sia-I^). In macrophages Raw264.7, we observed a band of the same molecular weight as the above mentioned for ST8Sia-I from SK-MEL-28 cells (Figure 1A), suggesting that ST8Sia-I is expressed in macrophage cells.

ST8Sia-I is a type II transmembrane protein localized mainly at the proximal Golgi complex, although a fraction is also located at the Trans-Golgi Network (TGN) and functionally coupled to N-acetylgalactosaminyltransferase [30]. In order to further evaluate the presence and subcellular localization of ST8Sia-I in macrophages, we performed an immunofluorescence colocalization analysis with specific Golgi complex markers (Manders’ Overlap Coefficient - MOC- was used as a measure of colocalization). Under permeabilized conditions, the results showed that endogenous ST8Sia-I from RAW264.7 cells have a perinuclear localization with high MOC with the cis-Golgi complex marker GM130 and low MOC with TGN38 protein (resident integral membrane protein of the TGN), in agreement with previous findings (Figure 1B). Then, the presence of ST8Sia-I at the plasma membrane of macrophages cells was studied under non-permeabilized conditions. Briefly, CHO-K1, CHO-K1^ST8Sia-I^ and RAW264.7 cells were first incubated at 4 °C to inhibit intracellular transport and then with antibody to ST8Sia-I at 4 °C for 60 min to allow membrane binding. Next, cells were fixed and processed for immunofluorescence. As shown in Figure 1C, ST8Sia-I was localized at cell surface of both cellular types. In CHO-K1^ST8Sia-I^ cells, if an anti-HA antibody is used instead of antibody to ST8Sia-I, the enzyme is also detected at the plasma membrane. In addition, GD3 ganglioside, which is mainly localized on the plasma membrane, was also detected at the cell surface of RAW264.7 cells under the experimental condition mentioned above (Figure S1). As expected, GD3 colocalized with ecto-ST8Sia-I, reinforcing the notion that ST8Sia-I is localized at cell surface. Furthermore, ecto-ST8Sia-I also displayed a similar localization as CD40, a plasma membrane glycoprotein (Figure S1). As a control, we observed that CHO-K1 cells did not bind antibody to ST8Sia-I (Figure 1C). Thus, these results strongly support the presence of ecto-ST8Sia-I at the cell surface of RAW264.7 macrophages.

Finally, we evaluated the cis-catalytic activity of ecto-ST8Sia-I at the plasma membrane of macrophages cells. To do this, we used a very sensitive and specific ELISA procedure previously validated and described to measure GD3 [22,23]. RAW264.7 cells were treated with P4 (a potent inhibitor of ceramide glucosyltransferase) to reduce GM3, GD3, and the neutral glycolipid content for 4 days (Figure 1D, see +P4, left panel). Then, cells were incubated with 25 μM GM3, washed and incubated in a medium containing Cytidine-5’-monophospho-N-acetylneuraminic acid (CMP-NeuAc), Mn^+2^, and Mg^+2^ at 37 °C for 60 min in the presence of P4 inhibitor. ecto-ST8Sia-I activity was determined by immunodetection of the synthesized GD3 product using the specific monoclonal antibody R24. As shown in Figure 1D (left panel, black bars), GD3 synthesis was detected at the cell surface of P4-treated RAW264.7 cells, indicating that ecto-ST8Sia-I was able to use the exogenously incorporated GM3 to catalytically convert it to GD3 ganglioside at the cell surface. In contrast, there was non-detectable amount of GD3 when P4-treated RAW264.7 cells were fed with GM3 and incubated in DMEM (+P4+GM3) only (culture medium containing 0.814 mM Mg^+2^) (Figure 1D, left panel, grey bars). In addition, the ELISA signals obtained from microtitration plates incubated with GM3, CMP-NeuAc, Mn^+2^, and Mg^+2^ were very weak, indicating the specificity of R24 antibody binding and consequently not significantly contributing to the positive signal in the experimental conditions analyzed (Figure 1D, right bar graph, left column). As expected, P4-untreated cells showed high levels of GD3-immnoreactivity (Figure 1D, right bar graph, right column). These results were further supported by using an experimental protocol combined with confocal microscopy analysis to measure ecto-ST8Sia-I activity [22,23]. Briefly, RAW264.7 cells grown on coverslips with P4 (+P4) were fed for 2 h with 25 μM GM3 before being washed and incubated at the same experimental conditions as mentioned above. Finally, GD3 was immunodetected using antibody R24 confirming the synthesis of GD3 at the cell surface (Figure 1E). Similar to ELISA assays, the incubation of macrophages with the two cofactors and the exogenous administration of the sugar nucleotide, significantly increased the synthesis of GD3. Thus, these results strongly suggest the presence of ecto-ST8Sia-I in macrophages cells and that ecto-ST8Sia-I localized at cell surface is able to synthesize GD3. It is worth mentioning that cells fed with GM3 and incubated with the cofactors Mn^2+^ and Mg^2+^ in the absence of exogenous CMP-NeuAc, a significant increase of GD3 synthesis was observed compared with P4-treated cells fed with GM3 and incubated only in DMEM (Figure S2), suggesting that cells can supply endogenous CMP-NeuAc to synthesize GD3.

### 2.2. Expression and Cis-activity of Ecto-ST8Sia-I in Macrophages Stimulated by LPS 

**Figure 2:**
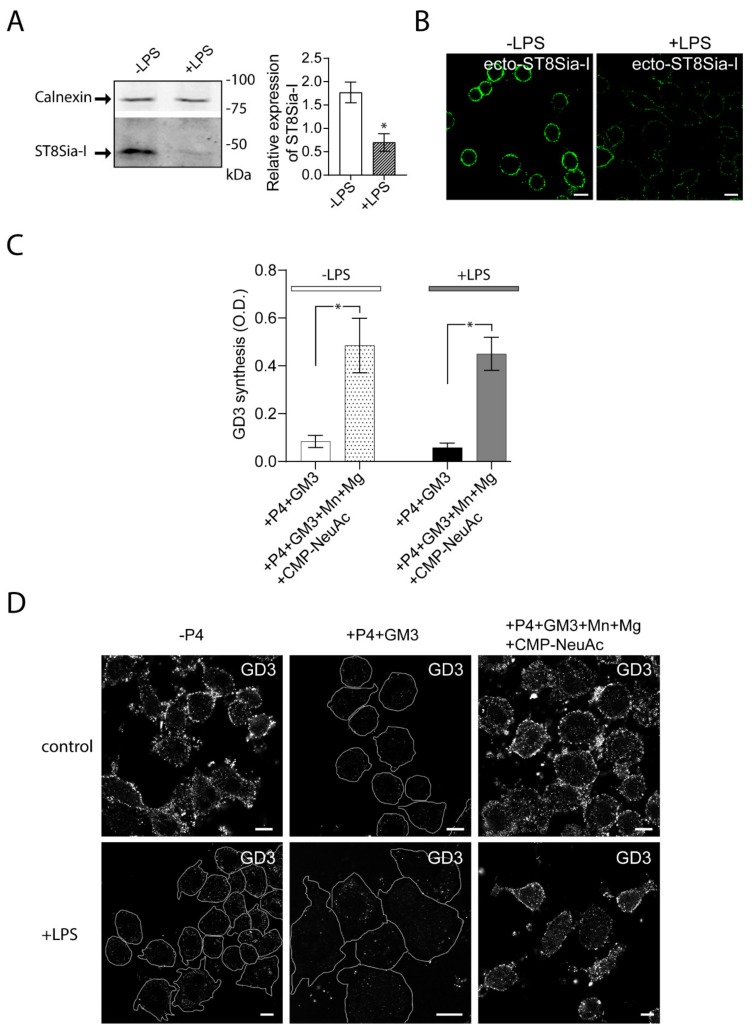
Reduced expression of ecto-ST8Sia-I and cis-activity of ecto-ST8Sia-I in Lipopolysaccharide (LPS) -stimulated macrophages. (**A**) Cell lysates of RAW264.7 cells stimulated (+LPS) and non-stimulated (-LPS) with LPS for 48 h were analyzed by Western blot using an antibody specific for ST8Sia-I. Calnexin was used as a loading control. Data are representative for three independent experiments. Quantification of relative expression of ST8Sia-I is shown (bar graph; * *p* < 0.05). (**B**) Cells stimulated (+LPS) and non-stimulated (-LPS) with LPS were immunostained with anti-ST8Sia-I antibody at 4 °C for 60 min. Then, cells were fixed, incubated with secondary antibody and visualized by confocal microscopy. (**C**) RAW264.7 cells treated with P4 for 3 days were stimulated with LPS for 48 h. Then, cells were treated with 25 µM GM3, washed, and incubated at 37 °C in a medium containing only DMEM (+P4+GM3) or in a medium containing CMP-NeuAc, Mn^+2^ and Mg^+2^. The P4 inhibitor remained present throughout the experiments. After 60 min, cells were washed and the GD3 synthesis for all experimental conditions was detected by ELISA as described in Figure 1 and in the Materials and Methods section. Note that GD3 synthesis was significantly higher in the medium containing exogenous CMP-NeuAc plus cations than in the medium containing only DMEM. Results are means ± SEM of two independent experiments. One-way ANOVA: F = 11.27, *p* < 0.005. Tukey’s multiple comparison test (* *p* < 0.05). (**D**) RAW264.7 cells grown with P4 (+P4) or without P4 (-P4) for 3 days were stimulated with LPS during 48 h. Then, cells were treated with 25 µM GM3, washed, and incubated at 37 °C for 60 min in a medium containing only DMEM (+P4) or containing CMP-NeuAc, Mn^+2^ and Mg^+2^ (+P4+Mn+Mg+CMP-NeuAc). The P4 inhibitor remained present throughout the experiments. Then, cells were washed, immunostained with antibody to GD3, fixed and incubated with secondary antibody conjugated to Alexa488. Representative confocal microscopy sections of 0.8 μm taken parallel to the coverslip are shown. Cell boundaries (white lines) are indicated. The fluorescence micrographs are representative of three independent experiments. Scale bar: 10 µm.

Then, we evaluated the impact of LPS stimulation of Raw264.7 cells on the expression and cis activity of ecto-ST8Sia-I. To do this, RAW264.7 cells were stimulated with LPS (100 ng/mL) for 48 h and then the expression of ecto-ST8Sia-I was analyzed by Western blot. The inflammatory stimulus resulted in a reduction of ecto-ST8Sia-I levels compared to control condition (Figure 2A). In agreement with this finding, confocal microscopy analysis showed low levels of ST8Sia-I at the plasma membrane respective to unstimulated cells (Figure 2B). Enzymatic cis-catalytic activity performed by ELISA showed that, when exogenous GM3 substrate was provided, ecto-ST8Sia-I was able to synthase GD3 ganglioside even in the presence of LPS (Figure 2C), indicating that macrophage activation by LPS modifies ST8Sia-I expression but not its enzymatic activity. Moreover, taken together with the results obtained by confocal microscopy (Figure 2D), our findings show that even minor levels of enzyme at the cell surface are sufficient to modify exogenous GM3 ganglioside.

### 2.3. The Alteration in ST8Sia-I Expression Correlates with a Reduction of GD3 and GM1 and with an Increment of GD1a at the Plasma Membrane

**Figure 3:**
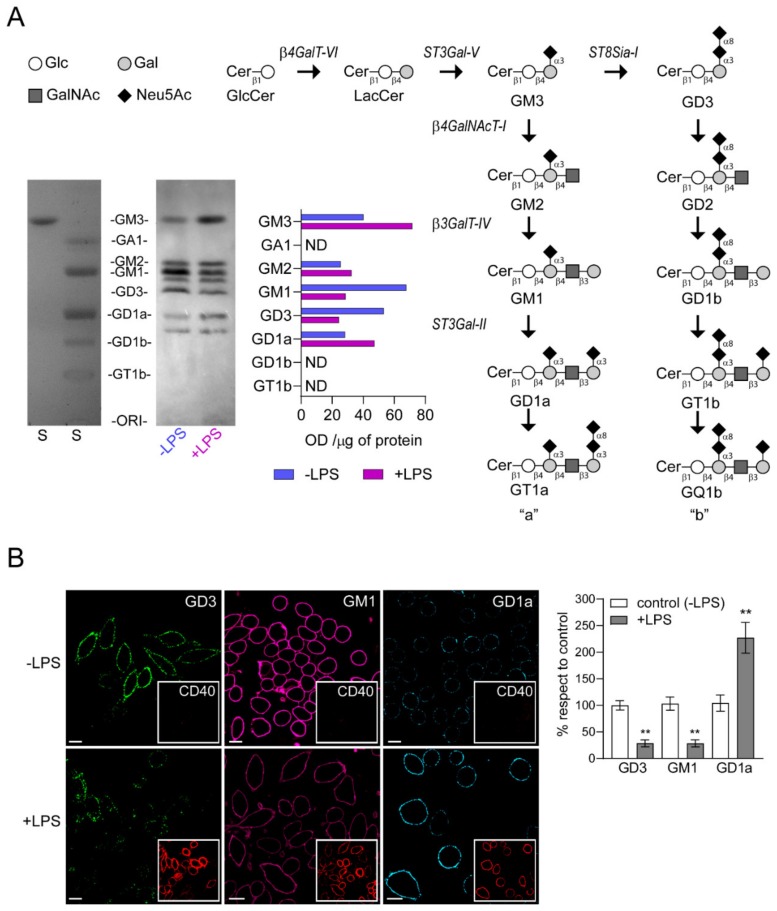
Change in the expression of gangliosides in RAW264.7 cells stimulated by LPS. (**A**) Pathway for ganglioside biosynthesis representing the stepwise addition of monosaccharides to ceramide, and the resulting structures. β4GalT-VI, UDP-Gal:glucosylceramide galactosyltransferase; ST3Gal-V, CMP-NeuAc:lactosylceramide sialyltransferase; ST8Sia-I, CMP-NeuAc:GM3 sialyltransferase, and CMP-NeuAc:GD3 sialyltransferase; β4GalNAcT-I, UDP-GalNAc:lactosylceramide/GM3/GD3/GT3 N-acetylgalactosaminyl transferase; β3GalT-IV, UDP-Gal:GA2/GM2/GD2/GT2 galactosyltransferase; ST3Gal-II, CMP-NeuAc:GA1/GM1/GD1b/GT1c sialyltransferase. Cer, ceramide; Glc, glucose; Gal, galactose; GalNAc, N-acetylgalactosamine; Neu5Ac, N-acetylneuraminic acid (sialic acid). Cells in culture stimulated (+LPS, red) and non-stimulated (-LPS, blue) with LPS were labeled with [9,10(n)-3H]palmitic acid during 40 h. The palmitic acid was added after 8 h of LPS stimulation. Next, lipid extracts were purified, resolved by High-performance thin-layer chromatography (HPTLC), and visualized as indicated under Materials and Methods section. Bands in the film quantified by densitometry using ImageJ software (NIH, USA). Optical Density (OD) values are expressed per μg of protein. (Glycolipid standards (S) were also co-chromatographed and visualized by exposing the plate to iodine vapor are indicated on the left of the plate. ND, not detected. **B**) Analysis of GD3, GM1 and GD1a expression at cell surface of LPS-stimulated macrophages. The cells were immunostained with CTxβ (which binds to GM1) or specific antibodies against the respective gangliosides at 4 °C for 60 min. Then, cells were fixed, incubated with secondary antibodies and visualized by confocal microscopy (GM1 pseudocoloured magenta and GD1a pseudocoloured cyan). Insets, anti-CD40 PE-conjugated was used as control of stimulated macrophages. Ganglioside content was analyzed by quantifying its fluorescence intensity using ImageJ software. Data are expressed as mean ± S.D. One-way ANOVA: F = 141.7, *p* < 0.005. Tukey’s multiple comparison test (** *p* < 0.005). (as a percentage with respect to control, non-stimulated cells). Scale bars:10 μm.

A high-performance thin layer chromatography (HPTLC) analysis was conducted to study the expression of gangliosides in LPS-stimulated cells. As shown in Figure 3A, gangliosides identified and expressed in control RAW264.7 cells (-LPS) include GM3, GM2, GM1, GD3 and GD1a. The expression levels of GM3, GM2 and GD1a were near 1.8, 1.3 and 1.7-fold higher, respectively, in LPS-stimulated group than in non-stimulated RAW264.7 cells. In contrast, there was a reduction of GM1 (about 0.4-fold) and GD3 (about 0.5-fold) expression, indicating that the ganglioside pattern is modified during the activation process of macrophages.

CD40, a costimulatory molecule for antigen presentation by immune cells, is expressed constitutively at relatively low levels on non-stimulated macrophages. In addition, several reports showed that LPS is a strong inducer of CD40 expression in RAW264.7 cells [31,32]. Therefore, we used the levels of CD40 as a marker to distinguish between stimulated and non-stimulated macrophages and analyzed the expression of GM1, GD3 and GD1a gangliosides at plasma membrane of LPS-stimulated cells (Figure 3B). RAW264.7 cells were stimulated with LPS (100 ng/mL) for 48 h and then the levels of GD3 and GD1a gangliosides were visualized by confocal microscopy using specific antibodies [23,33]. GM1 ganglioside was detected using the cholera toxin subunit B (CTx-B), which has high affinity for GM1. We observed a reduction in both GD3 and GM1 gangliosides levels and an increase of GD1a after LPS stimulation of macrophages when compared to the control condition. In addition, the levels of CD40 expression on stimulated macrophages correlated with the ganglioside variations at the cell surface. Altogether, these findings demonstrate that the decrease in ecto-ST8Sia-I expression after LPS-stimulated macrophages correlate with a reduction of its product GD3, a decrease of GM1 and an increase of GD1a.

### 2.4. Relative Levels of NEU1, NEU3 and ST3Gal-II Transcripts Analyzed by RT-qPCR

**Figure 4:**
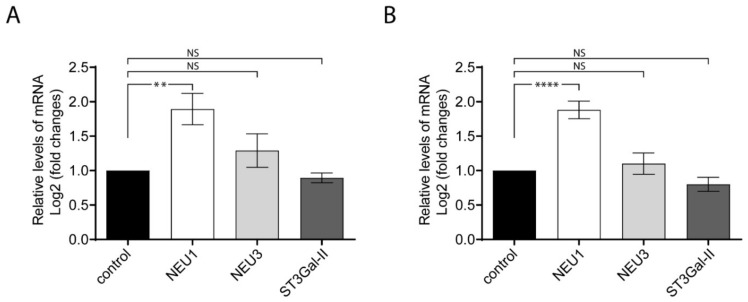
The relative levels of NEU1, NEU3 and ST3Gal-II transcripts analyzed by RT-qPCR. Total RNA was purified and reverse-transcribed from RAW264.7 cells stimulated and non-stimulated with LPS. mRNA levels were quantified by RT-qPCR using specific primers. The expression values for the RT-qPCR are given relative to the expression levels of 18S rRNA and Tbp (TATA-box-binding protein) gene (**A** and **B**, respectively) and were expressed as Log2 fold change. Data are expressed as mean ± S.E.M. from at least three independent experiments, each in triplicate. (** *p* < 0.005; **** *p* < 0.0001).

The sialylation process is generally maintained on the activity of sialyltransferases and sialidases. The variations of gangliosides expression described above (Figure 3) suggest that other enzymes associated to gangliosides metabolism could be modified by an inflammatory stimulus. In particular, we evaluated by qPCR in LPS-treated cells the gene expression of ST3Gal-II, the main sialyltransferase responsible for GD1a ganglioside biosynthesis; sialidase NEU3, a key enzyme in the catabolism of gangliosides like GD1a, GD3 and GM3; and the sialidase NEU1, which predominantly uses glycoproteins as substrates. The calculation of the relative expression of a target-gene by qPCR was carried out using two reference genes as endogenous controls (18S rRNA and Tbp for Figure 4A,B, respectively). The results showed that ST3Gal-II and NEU3 remained without changes after LPS-stimulated cells. On the contrary, NEU1 was upregulated after LPS-stimulation, in agreement with recent findings [28,34]. The data indicate that proinflammatory macrophage stimulation activates the expression of selective sialidases and sialyltransferases.

### 2.5. NO Formation is Involved in the Expression/Localization of ST8Sia-I at the Plasma Membrane

**Figure 5:**
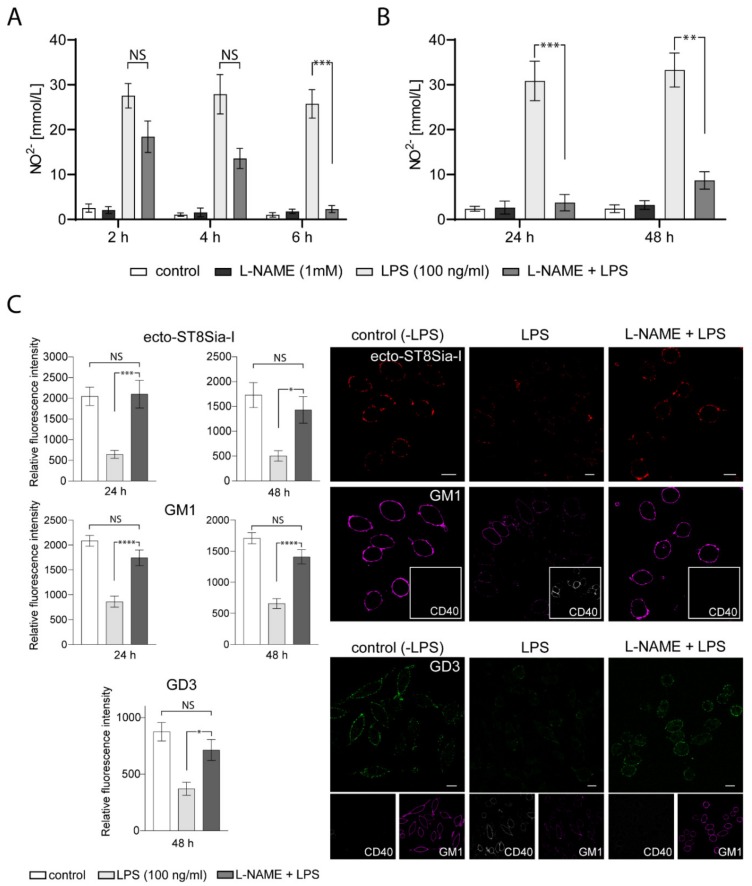
Effect of L-NAME on ST8Sia-I levels at the plasma membrane of LPS-treated macrophages. (**A**) Four experimental groups were used: Cells untreated (control), cells pretreated with 1 mM L-NAME (L-NAME), cells stimulated with LPS (LPS 100 ng/mL) for 24 h and cells pretreated with 1 mM L-NAME and stimulated with LPS (L-NAME+LPS) at the indicated times. The Nitric oxide (NO) was measured using Griess assay. Data is representative of two independent experiments. One-way ANOVA: F = 14.87, *p* < 0.0001. Tukey’s multiple comparison test. (**B**) The same experimental groups described in **A** were used, except that cells were stimulated with LPS for 24 h and 48 h. The NO was measured using Griess assay. Data is representative of three independent experiments. One-way ANOVA: F = 22.27, *p* < 0.0001. Tukey’s multiple comparison test. (**C**) Cells untreated (control), cells stimulated with LPS (LPS 100 ng/mL) and cells pretreated with 1 mM L-NAME and stimulated with LPS (L-NAME+LPS) were immunostained with CTxβ (which binds to GM1) and specific antibodies against the respective sialyltransferase ST8Sia-I, GD3 ganglioside and glycoprotein CD40 at 4 °C for 60 min. Then, cells were fixed, incubated with secondary antibodies and visualized by confocal microscopy. Anti-CD40 PE-conjugated was used as the control of stimulated macrophages. Where indicated, cells were stimulated with LPS for 24 h or 48 h. (ST8Sia-I pseudocoloured red, GM1 pseudocoloured magenta, GD3 pseudocoloured green and CD40 pseudocoloured grey). The levels of ST8Sia-I, GM1 and GD3 were analyzed by quantifying its fluorescence intensity using ImageJ software. Data are expressed as mean ± S.E.M. One-way ANOVA and Tukey’s multiple comparison test were used. (* *p* < 0.05; ** *p* < 0.005; *** *p* < 0.0005; **** *p* < 0.0001). Scale bars:10 μm.

Many studies indicate that gangliosides may interact with components of the inflammatory response pathway such as NO [35,36,37]. In macrophages, it has been suggested that the inhibitory action of exogenous gangliosides on these cells is through the modulation of inducible NO synthase (iNOS) expression [38]. However, we did not find any reports on the ability of NO to modify the levels of endogenous sialyltransferases, specifically ecto-ST8Sia-I. To evaluate this possibility, we tested the effect of NG-nitro-L-arginine methyl ester (L-NAME), a competitive inhibitor of NO synthesis, in LPS-stimulated RAW264.7 macrophages. First, we tested the effect of L-NAME incubation previous to LPS treatment. As shown in Figure 5A, a significant reduction of NO production resulted from preincubation with L-NAME (2 mM) for 6 h before adding LPS for 24 h. Furthermore, NO inhibition by L-NAME persisted 48 h after LPS-stimulation (Figure 5B). Interestingly, L-NAME treatment of LPS-stimulated macrophages for 24 h or 48 h prevented the reduction of ecto-ST8Sia-I expression and reversed the decrease in GM1 levels observed in LPS-stimulated macrophages (Figure 5C). Moreover, GD3 levels also remained without significative changes in macrophages incubated with L-NAME previous to LPS treatment for 48 h (Figure 5C). In addition, CD40 expression levels were not affected by LPS after L-NAME treatment, indicating the effectiveness of the procedure in inhibiting macrophage activation. These results strongly suggest that NO production is involved in the variations of endogenous ecto-ST8Sia-I expression and gangliosides levels produced by activation of macrophages by LPS.

## 3. Discussion

Sialyltransferases and sialidases are two important players in the sialylation of glycoproteins and glycolipids on the surface of diverse cellular types. In addition to the presence of glycohydrolases and sialidases associated with the outer layer of the plasma membrane, we demonstrated in epithelial and melanoma cells that sialyltransferase ST8Sia-I is able to sialylate GM3 ganglioside exposed on cell surface itself or exposed at the plasma membrane of neighboring cells [22,23]. To the best of our knowledge, this is the first report that shows the presence of ecto-ST8-I at the plasma membrane of macrophages, besides its classical localization at the Golgi complex. Moreover, we demonstrated that this sialyltransferase was able to sialylate exogenous GM3 ganglioside incorporated at the plasma membrane. The proinflammatory stimulation of macrophages with LPS produced a decrease of ecto-ST8Sia-I expression concomitant with a reduction of GD3 and an increase of GM3 gangliosides (Figure 3). The de novo synthesis of GM3 and GD3 is carried out in the Golgi complex by the successive addition of galactose and then sialic acid residues on glucosylceramide by the enzymatic complex formed by β4GalT-VI, ST3Gal-V and ST8Sia-I, respectively (Figure 3) [39]. ST8Sia-I works as a polysialyltransferase that uses GM3 more efficiently than GD3 to synthesize GD3 and GT3, respectively [40]. At steady-state, the stable expression of ST8Sia-I in wild-type CHO-K1 cells, which express predominantly the ganglioside GM3, synthesize mostly GD3 and GT3 and practically no GM3 is detected [41], indicating that the majority of GM3 produced by cells is used to synthetize GD3 when ST8Sia-I is present. Also, CHO-K1 cells genetically modified to express a-series complex gangliosides [42], accumulate appreciable amounts of GM3, indicating that GM3 is not fully used for the synthesis of GM2, GM1 and GD1a. Thus, the increment of GM3 in LPS-stimulated macrophages could be due to the low levels of ST8Sia-I available to form the multi-enzyme complex. Earlier investigations [43] showed in primary rat astrocytes that LPS/interferon-γ (IFN-γ) treatment increases the activity of β4GalT-VI (lactosylceramide synthase, Figure 3), the intracellular levels of lactosylceramide and induced iNOS gene expression. In addition, the treatment with an inhibitor of glucosylceramide synthase and lactosylceramide synthase activities reversed the effect of LPS. Therefore, the increment in the activity of β4GalT-VI in the inflammatory response may also contribute to the high levels of GM3 expression after LPS stimulation in macrophages. The gangliosides pattern in LPS-treated cells also showed an increment of GD1a with a concomitant decrease of GM1, product and substrate of ST3Gal-II, respectively. These findings suggested that ST3Gal-II levels are modified by LPS treatment. However, mRNA expression of ST3Gal-II remained unaltered (Figure 4). One possible explanation of these results is that GM3 accumulation can be exploited by the complex formed by β4GalNAcT-I and β3GalT-IV [44] and then by ST3Gal-II to produce more GD1a in activated macrophages. Several reports have described that complex formation between glycosyltranferases improve the enzymatic activity of one of the partners [45,46]. Therefore, a complex formed by β4GalNAcT-I, β3GalT-IV and ST3Gal-II, which improved the ST3Gal-II activity, emerges as a plausible candidate to produce more efficiently GD1a ganglioside.

Two of the four mammalian sialidases characterized [47,48,49,50], NEU3 and NEU1, enhances LPS-induced cytokine production in primary human monocyte-derived cells [51]. NEU3, a membrane-bound protein associated with the plasma membrane and endosomes [52], desialylates more efficiently GD3, GM3 and GD1a than GM2 and GM1. In this study, it was observed that mRNA levels of NEU3 are not modified by LPS stimulation, suggesting that NEU3 is not involved with the variations in the gangliosides pattern of activated macrophages. On the other hand, NEU1, expressed in lysosomes and the surface of diverse types of cells and preferentially desialylates glycoproteins [53], is upregulated in macrophages stimulated with LPS. This result agrees with a recent investigation describing the role of NEU1 in the modulation of TLR4 sialylation [28]. Besides, NEU1 can also be targeted to the cell surface under inflammatory activation of macrophages [54].

Here, the LPS stimulation of macrophages also reduced the expression of ST8Sia-I at the plasma membrane. Despite this matter, ecto-ST8Sia-I was able to sialylate exogenous GM3 at the same level than non-stimulated cells. One possible explanation is that exogenous GM3 could be endocytosed and processed by ST8Sia-I resident in the Golgi. However, previous work by our laboratory showed that impairing plasma membrane internalization by treatment with impermeable fixative tannic acids did not affect synthesis of GD3 at the cell surface of CHO-K1^ST8Sia-I^ cells, although transferrin endocytosis was severely affected [22]. This discards the involvement of internal ST8Sia-I and supports our present findings of surface, ecto-ST8Sia-I activity. In addition, our results provide evidence of the capacity of macrophages, under pro-inflammatory conditions, to reduce the expression of ST8Sia-I and its product GD3, maintaining minimal levels of the enzyme at the plasma membrane to quickly respond towards different external stimuli. In this context, exogenous GD3 rapidly associates with the cell membrane of human arterial smooth muscle cells, recruits reactive oxygen species such as superoxide and NO (in a dose-dependent manner) as biological sensors that serve a dual role in cell proliferation and apoptosis, respectively [55]. On the other hand, the authors describe that GM3 reduces the cellular level of superoxide. Therefore, the presence of ST8Sia-I at the cell surface may offer an additional route of rapid control of GD3 and GM3 levels at the plasma membrane and modulating, in consequence, the production reactive oxygen species like superoxide and NO. Cell surface gangliosides sialylation, first reported in synaptosomal membrane [56,57], has also been reported in other cell types such as human brain-derived TE671 cells [58] and polymorphonuclear leukocytes, which express the sialidase and sialyltransferase activities that allow a rapid modulation of their surface sialylation and hence adhere to and migrate across the endothelium [59].

The control of the relative levels of ST8Sia-I in Golgi versus plasma membrane and the specific impact of LPS on these two populations is largely unknown and deserves further investigation. Several reports describe alteration of glycosylation after LPS stimulation in macrophages [60,61]. It is well known that ST8Sia-I localized at the proximal Golgi, contains three *N*-glycosylation sites occupied by *N*-glycans [30,62]. In addition, pharmacological and biochemical assays of the N-glycan status of ecto-ST8Sia-I strongly suggest that it might arrive at the plasma membrane from the medial Golgi through a secretory trans-Golgi network bypass route or directly by a Golgi-independent trafficking, as previously suggested for both polarized and nonpolarized cells [63,64]. Thus, glycosylation changes triggered by LPS stimulation could affect stability of ST8Sia-I and promote proteolytic degradation leading to a reduction in Golgi compartment and also at the plasma membrane. 

NO is an important mediator in innate immunity with the ability to participate in diverse regulatory and cytotoxic actions [65,66]. Here, we uncovered that LPS leads to upregulation of NO, shedding light into the possible mechanism linking LPS-mediated activation of macrophages and reduction of ST8Sia-I levels. We determined that endogenous NO may interfere with the variations of endogenous ecto-ST8Sia-I and several gangliosides levels produced by stimulation of macrophages with LPS (Figure 5). It is known that NO and its derivatives can elicit nitration of tyrosine residues and S-nitrosylation of cysteine residues in target proteins [67]. In LPS-stimulated macrophages, the increase of NO is followed by formation of protein-bound 3-nitrotyrosine, the most common protein tyrosine nitration [68]. Tyrosine nitration is a selective process [69] that may affect protein structure and cause protein dysfunction or alterations in signal transduction pathways, which includes changes in the rate of proteolytic degradation [70]. At the present, there is no experimental data indicating the existence of tyrosine nitration in ganglioside sialyltransferases or sialidases. Thus, using GPS-YNO2 and iNitro-Tyr softwares [71,72], two described 3-nitrotyrosine prediction algorithms, a set of predictions for nitrated residues in ST8Sia-I and others ganglioside sialyltransferases and sialidases was generated (Table S1). In total, ST8Sia-I have 16 tyrosine residues. GPS-YNO2 predicted one tyrosine nitration (Y181) and iNitro-Tyr software predicted three tyrosine nitration (Y212, Y320 and Y332). Taking these predictions into consideration, we propose that the increase of NO and its derivatives in activated macrophages might result in a subsequent nitration of the tyrosine residues of ST8Sia-I leading to degradation of the enzyme and, in consequence, a reduction in the levels of the protein. Much research remains to be done on this topic to determine the molecular events that regulate the endogenous expression of gangliosides and sialyltransferases by NO.

## 4. Materials and Methods

### 4.1. Cell Culture and Stimulation

CHO-K1 cell clones expressing different ganglioside glycosyltransferases had been obtained previously in our laboratory. The following cells were used: wild-type CHO-K1 cells (ATCC, Manassas, VA, USA); clone 2 (CHO-K1^ST8Sia-I^), a stable chick ST8Sia-I (tagged at the C terminus with the YPYDVPDYA nanopeptide epitope of the viral HA) transfectant expressing the gangliosides GD3 and GT3 [6,30]. SK-Mel 28 human melanoma cell line (ATCC), which endogenously expresses ST8Sia-I. Cells were grown and maintained at 37 °C in 5% CO_2_ in DMEM supplemented with 10% FBS. RAW264.7 murine macrophages (ATCC) were maintained in DMEM with 5% FBS, 2 mM L-glutamine. RAW 264.7 macrophages were grown to 60–70% confluency before stimulation. Cells were stimulated with 100 ng/mL LPS for 24 or 48 h (from Escherichia coli 0111:B4, Cat.# L2630, Sigma, Saint Louis, MO, USA). Where indicated, cells were pretreated with L-NAME (1 mM; Sigma, St. Louis, MO, USA) at different times before adding LPS to the cells. L-NAME was kindly provided by Dr. Cinthia Stempin (Centro de Investigaciones en Bioquímica Clínica e Inmunología, CIBICI, Universidad Nacional de Córdoba, Argentina).

### 4.2. Assay of NO Production

NO released from cells in culture was quantified by measurement of the NO metabolite, nitrite. After stimulation, 50μL of culture medium was mixed with 50 μL sulfanilamide (1%) in 0.5 N HCl. After a 5-min incubation at room temperature, an equal volume of 0.02%N-(1-naphthyl)-ethylenediamine was added. Following incubation for 10 min at room temperature, nitrite levels were determined using sodium nitrite as a standard.

### 4.3. Antibodies

The following antibodies were used: monoclonal mouse antibody to GD3 (clone R24; ATCC); monoclonal mouse antibody to GD1a (kindly supplied by *p*.H. Lopez, INIMEC-CONICET, Cordoba, Argentina); monoclonal mouse antibody to tubulin (Sigma-Aldrich, Saint Louis, MO, USA); monoclonal rabbit antibody to GD3 Synthase Antibody (H-76, Santa Cruz Biotechnology, Santa Cruz, CA, USA); anti mouse CD40 conjugated with Phycoerythrin (PE) (BioLegend, San Diego, CA, USA); mouse monoclonal anti-GM130 (BD Biosciences); mouse monoclonal antibody anti-TGN38 (Abcam Inc., Cambridge, MA, USA); monoclonal mouse antibody to HA epitope (Sigma-Aldrich, St Louis, MO, USA); polyclonal rabbit antibody to Calnexin (Abcam Inc.); TruStain FcX (anti-mouse CD16/32 antibody, clone: 93, BioLegend).

The secondary antibodies used were Alexa Fluor® 488-conjugated goat anti-mouse IgG, Fluor® 488-conjugated goat anti-rabbit IgG, Alexa Fluor® 546-conjugated goat anti-mouse IgG, Alexa Fluor® 546-conjugated goat anti-rabbit IgG (Invitrogen, Carlsbad, CA, USA) for immunofluorescence and goat anti-rabbit or mouse IgG coupled to IRDye800CW or IRDye600CW (LI-COR Biotechnology) for Western blotting. Cholera Toxin Subunit B (Recombinant), Alexa Fluor™ 647 Conjugate (CTx-β) was used to bind GM1 ganglioside.

### 4.4. GD3 Detection by ELISA

GM3 was purified in our laboratory from dog erythrocytes. GD3 purified from chicken brain and glycolipids standards were kindly provided by G. Nores (Centro de Investigaciones en Química Biológica de Córdoba, CIQUIBIC, Universidad Nacional de Córdoba, Argentina). Different amounts of GM3 (50 and 100 pmol) or GD3 (0.75, 1.5, 3.0, and 6.0 pmol) were coated on polystyrene microtitration plates in methanol and dried overnight at 37 °C. After ganglioside coating, wells were saturated with 2% BSA in PBS for 2 h. The reactivity to GD3 was measured using the specific mouse monoclonal antibody anti-GD3 (IgG3) clone R24 (ATCC no. HB-8445) [23]. After washes, primary R24 antibody was detected by incubating it overnight at 4 °C with mouse IgG antibody conjugated with HRP and diluted 1:1000 in 2% BSA/PBS. After more washes, HRP activity was revealed using 0.5 mg/mL o-phenylenediamine in 0.1 M citrate-citric acid buffer (pH 5.5) containing 0.03% H_2_O_2_. The reaction was stopped by addition of H_2_SO_4_ (0.5 M final concentration), and the optic density was measured at 490 nm in a microplate reader (Bio-Rad, model 680). The GD3 synthesis was calculated by subtracting the nonspecific GD3 binding from each measurement.

### 4.5. Determination of ecto-ST8Sia-I Activity at the Cell Surface

The ecto-ST8Sia-I activity was measured as described by Crespo et al. (2010) [23] with minor modifications. Briefly, RAW264.7 cells were grown on 96-well polystyrene flat-bottom plates with 2 μM of d,l-threo-1-fenyl-2-hexadecanoilamino-3-pirrolidino-1-propanol-HCl (P4) (Matreya, Inc., PA, USA) to reduce GM3, GD3, and the neutral glycolipid content. Where indicated, cells were stimulated with 100 ng/mL LPS for 48 h. Then, cells were incubated for 2 h with 25 μM GM3. Next, cells were washed repeatedly with 0.2% BSA in PBS to remove the GM3 and then incubated for 60 min in an incubation system containing 10 mM MnCl_2_, 1 mM MgCl_2_, 100 mM sodium cacodylate-HCl buffer (pH 6.5), and 25 μM CMP-NeuAc in a volume of 50 μl of DMEM. Finally, cells were washed and incubated with TruStain fcX to block Fc receptors for 10 min and then incubated with antibody to GD3 (R24) at 4 °C for 60 min. Then, cells were washed, fixed with 2% PFA in PBS for 10 min at room temperature and incubated at 4 °C with secondary antibody conjugated to HRP in 2% BSA/PBS. After washes, HRP activity was revealed using 0.5 mg/mL o-phenylenediamine in 0.1 M citrate-citric acid buffer (pH 5.5) containing 0.03% H_2_O_2_. The reaction was stopped by addition of H_2_SO_4_ (0.5 M final concentration) and optic density was measured at 490 nm. The GD3 synthesis was calculated by subtracting the nonspecific GD3 binding (optic density value from a well containing RAW264.7 cells treated with P4) from each measurement.

To detect ecto-ST8Sia-I activity by immunodetection of the synthesized GD3 using the specific mouse monoclonal antibody anti-GD3 clone R24, macrophages cells RAW264.7 were grown on 8-well chambered coverglass (Lab-Tek chambers) and treated for 4 days with 2 μM P4. Where indicated, cells were stimulated with 100 ng/mL LPS for 48 h. Then, cells were incubated for 2 h with 25 μM GM3. Later, cells were washed repeatedly with 0.2% BSA in PBS to remove the GM3 and then incubated for 1 h in an incubation system containing 10 mM MnCl_2_, 1 mM MgCl_2_, 100 mM sodium cacodylate-HCl buffer (pH 6.5), and 25 μM CMP-NeuAc in a volume of 80 μl of DMEM. Finally, coverslips were processed for immunodetection of the synthesized GD3 and confocal microscopy analysis.

### 4.6. Confocal Immunofluorescence Microscopy

Cells were seeded onto glass coverslips (8-well chambered coverglass-Lab Tek; 15.000 cells). Cells were fixed with 1% (w/v) PFA in PBS for 10 min. After washes with PBS, cells were incubated with primary and secondary antibodies. In some cases, cells were permeabilized with 0.1% Triton X-100/200 mM glycine in PBS for 2 min at room temperature. Before cell surface staining, cells were incubated with TruStain fcX to block Fc receptors for 10 min on ice. For staining of the plasma-membrane-associated gangliosides, ST8Sia-I and CD40 cells were incubated with the corresponding antibodies or with CTx-β (which binds to GM1) at 4 °C for 60 min before fixation.

Confocal images were collected using an Olympus FluoView FV1000 confocal microscope (Olympus Latin America, Miami, FL) equipped with an argon/helium/neon laser and a ×63 (numerical aperture = 1.4) oil immersion objective (Zeiss Plan-Apochromat). Single confocal sections of 0.8 μm were taken parallel to the coverslip (*xy* sections). Final images were compiled with Adobe Photoshop. The confocal fluorescence micrographs shown in this manuscript are representative of at least three independent experiments.

### 4.7. Electrophoresis and Immunoblotting

Cells homogenates were resolved by electrophoresis through 10% SDS-polyacrylamide gels under reducing conditions. Proteins were electrophoretically transferred to nitrocellulose membranes for 90 min at 300 mA. Nonspecific binding sites on the nitrocellulose membrane were blocked with 5% defatted dry milk in 200 mM NaCl and 50 mM Tris/HCl pH 7.5 (TBS), and incubated overnight at 4 °C with primary antibodies diluted in TBS containing 0.05% Tween 20 (TTBS). After three washes with TTBS, membranes were incubated with secondary antibodies diluted in TTBS for 2 h at room temperature. Bands of proteins were detected using an Odyssey infrared imaging system (LI-COR Biotechnology, Lincoln, NE, USA). Molecular masses were calculated based on calibrated standards run in parallel. Tubulin and calnexin were used as a loading control. The images were obtained using Odyssey Application Software version 2.1, with the final images being compiled using Adobe Photoshop.

### 4.8. Lipid Labeling, Extraction, and Chromatography

Metabolic labeling, lipid extraction and chromatography were performed mostly as described [73]. Cells in culture stimulated and not stimulated with LPS (3 × 10^5^ cells/35-mm dish) were labeled with 5 μCi/mL [9,10(*n*)-^3^H]palmitic acid (Amersham Biosciences; 53 Ci/mmol) during 40 h. The palmitic acid was added after 8 h of LPS stimulation. After incubation, cells were washed with phosphate-buffered saline (PBS), and lipids were extracted with chloroform:methanol (2:1, *v/v*) at room temperature for 60 min and to the extract 0.2 vol. of water was added to achieve a Folch partition. The upper phase was freed from water-soluble contaminants by being passed through a Sephadex G-25 column. The lipid extract was used for radioactivity quantification or for thin-layer chromatography (TLC) analysis, supplemented with the appropriate amounts of standard gangliosides, and chromatographed on high performance TLC plates (HPTLC; Merck) using C:M:0.2% CaCl_2_ (60:36:8 *v/v*) as solvent. Standard gangliosides were visualized by exposure of the plate to iodine vapors. Radioactive gangliosides were visualized using a Fuji Photo Film Bio Imagen analyzer or visualized by fluorography after dipping the plate in 0.4% melted 2,5-diphenyloxazole in 2-methylnaphthalene and exposing it to a radiographic film at −70 °C. Densitometric quantification of X-ray plates was done using ImageJ software (National Institute of Health, USA).

### 4.9. RNA Isolation and cDNA Synthesis 

Total RNA was purified using TRIzol® reagent (Invitrogen, Carlsbad, CA, USA) according to the manufacturer’s instructions. Total RNA (5 μg) was utilized as a template for the cDNA synthesis reaction using SuperScriptTM III Reverse Transcriptase (Invitrogen, Carlsbad, CA, USA) and a blend of oligo(dT) and random hexamers (Biodynamics, Buenos Aires, Argentina) according to the manufacturer’s instructions.

### 4.10. qPCR (Quantitative Real-time PCR) 

SYBR Green-based qPCR was achieved as reported previously [74]. Primers specific to CHO-K1 endogenous ST3Gal-II, NEU1 and NEU3 were designed and purchase from Invitrogen (Carlsbad, CA, USA). The quantifications were performed in Rotor-Gene Q equipment (Qiagen, Hilden, Germany). The amplification mixture contained 1 μl of the cDNA synthesis reaction, 0.8 mM of each primer and 7.5 μl of Real Mix (Biodynamics, Buenos Aires, Argentina) in a total volume of 15 μl. The cycling conditions were 30 s of polymerase activation at 95 °C and 40 cycles of 95 °C for 30 s, 60 °C for 30 s and 72 °C for 30 s. Each assay included a standard curve in duplicate, utilizing 1:5 serial dilutions of cDNA from RAW264.7 cells stimulated and not stimulated with LPS. Samples were measured in triplicate. The PCR product was checked by melt curve analysis, and the standard curve linearity and PCR efficiency were optimized. The data was analyzed with Rotor-Gene Q software (Qiagen, Hilden, Germany). The relative concentration values were normalized by the geometric average of two internal control genes: Tbp (TATA-box-binding protein) and 18S rRNA [75].

### 4.11. Statistical Analyses

Results are presented as means ±SD or ±S.E.M. Statistical analyses were made using Student’s t test or ANOVA with Prisma 8.3.0 (*) was attributed at the 95% level of confidence (*p* < 0.05).

### 4.12. Image Processing 

Final images were compiled with Adobe Illustrator CC 23.0.1, with the confocal fluorescence micrographs in the present paper being representative of at least three independent experiments. Scale bars in all figures represent 10 μm. Quantification of fluorescence intensity (arbitrary units/cell area) of a focal plane of at least 20 cells of each experimental condition was performed using ImageJ software (NIH). Manders’ colocalization coefficients were calculated using the JACoP ImageJ plugin with at least ten cells for each experimental condition being imaged on the *z*-axis (five z-slices).

## Supplementary Materials

Supplementary materials can be found at https://www.mdpi.com/1422-0067/21/3/1063/s1.
