# Supplementary material for: Ganglioside Synthesis by Plasma Membrane-Associated Sialyltransferase in Macrophages
Source: Int J Mol Sci. 2020 Feb 5;21(3):1063. doi: 10.3390/ijms21031063 (PMC7043224; doi:10.3390/ijms21031063)
Supplement: Supplementary file 1 [file ijms-21-01063-s001.zip › Supplementary File/Supplementary File.docx]

**Figure S1**


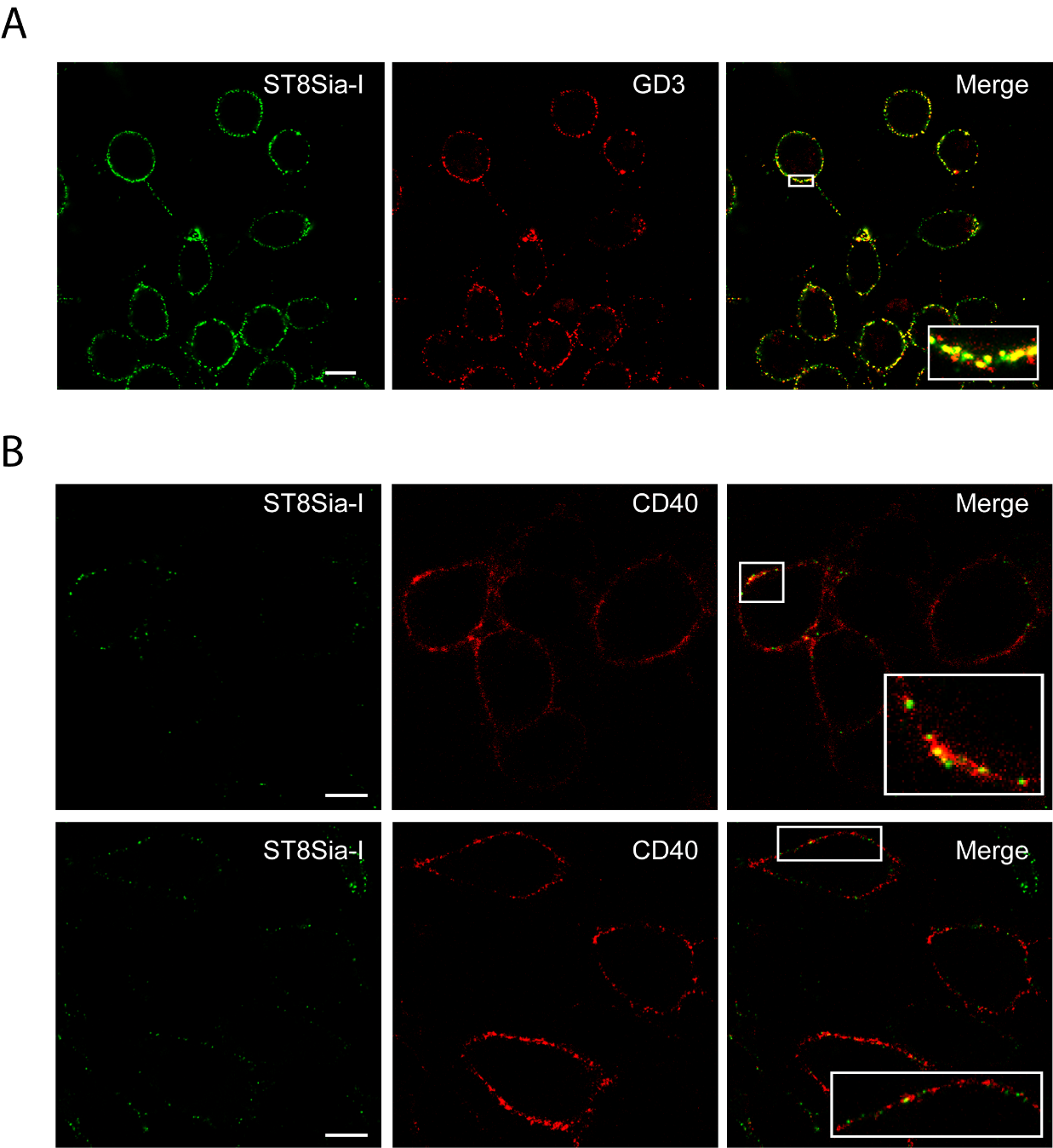


**Immunofluorescence detection of ST8Sia-I, GD3 and CD40 at cell surface of RAW264.7 cell line**. (**A**) Cells were immunostained with specific antibodies to ST8Sia-I and GD3 ganglioside at 4 °C for 60 min. Then, cells were ﬁxed, incubated with secondary antibodies and visualized by confocal microscopy. Insets show details at higher magniﬁcation. (**B**) Cells were stimulated with LPS (LPS 100 ng/ml) for 48 h and immunostained with specific antibodies against ST8Sia-I and glycoprotein CD40 at 4 °C for 60 min. Then, cells were ﬁxed, incubated with secondary antibodies and visualized by confocal microscopy. Insets show details at higher magniﬁcation. Scale bars:10 μm.

**Figure S2**

**
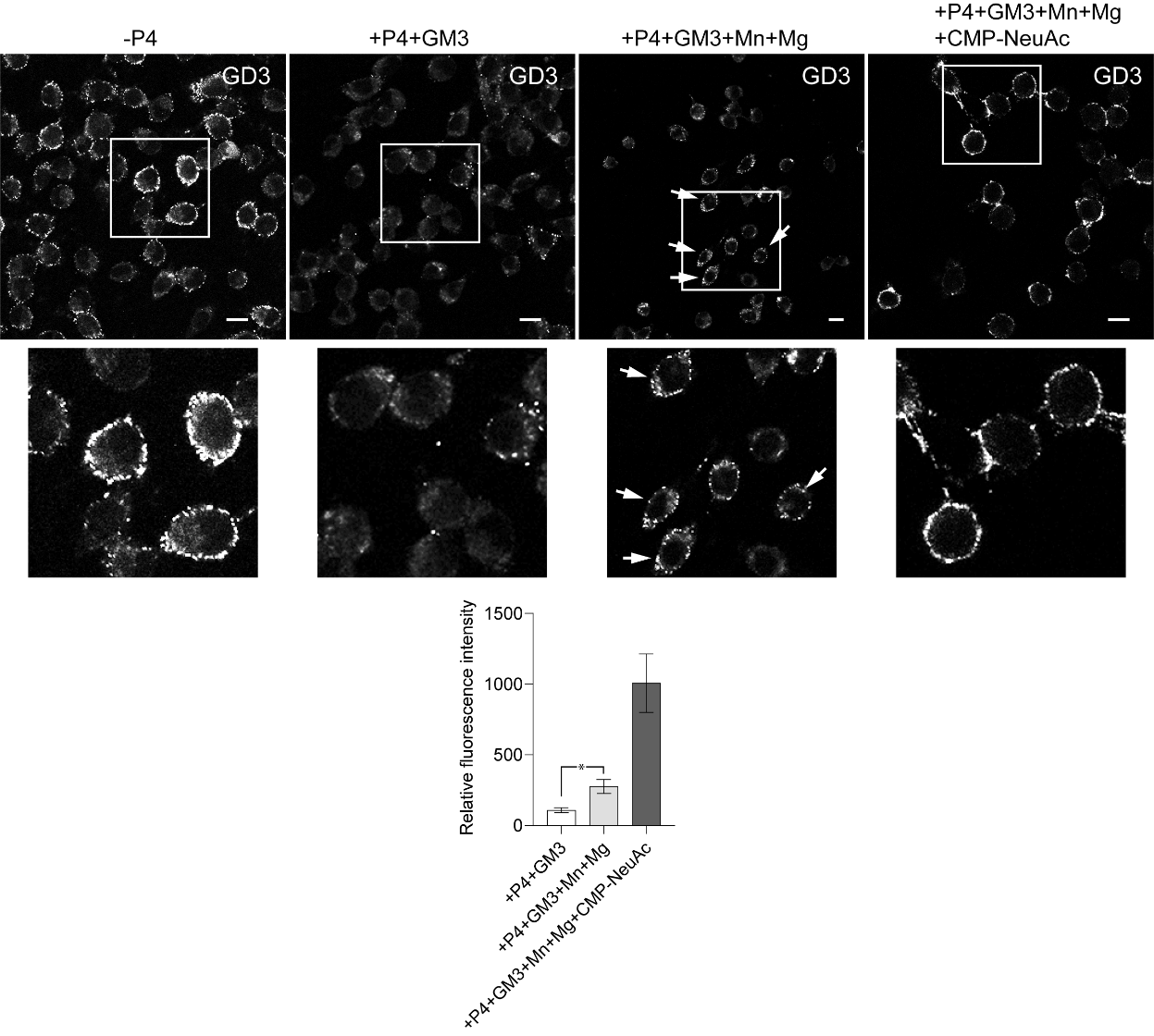
**

**Ecto-ST8Sia-I sialylates exogenously incorporated GM3 in RAW264.7 cells**. Macrophage RAW264.7 cells were grown with P4 or without P4 (−P4) for 4 days. Then, cells were treated with GM3, washed, and incubated at 37 °C for 1 h in a medium containing only DMEM (+P4+GM3) or in a medium containing Mn^2+^ and Mg^2+^ (+P4+GM3+Mn+Mg) or CMP-NeuAc, Mn^2+^ and Mg^2+^ (+P4+GM3+Mn+Mg+CMP-NeuAc). P4 inhibitor remained present throughout the experiments. Cells were washed, immunostained with antibody to GD3 at 4 °C for 60 min, and then fixed and incubated with secondary antibody conjugated to Alexa Fluor 488. Rows indicate GD3 synthesis. Insets show details at higher magniﬁcation. Single confocal sections were taken every 0.7 μm parallel to the coverslip. Scale bars:10 μm.

**Table S1.** Nitration sites in the listed proteins from mouse were computationally predicted using GSP-YNO2 1.0 and iNitro-Tyr software. “*” non-predicted site. “n.d.” not determined. P60710 (actin, cytoplasmic 1), which nitrated Tyrosine residues have been identified experimentally (see references below) was using as cross-validation (please, see references [1, 2] for further information). ∼ 95 % of the total number of tyrosine nitration sites were successfully indicated by the algorithms. 53 [3, 4], 69 [5], 91 [6], 169 [6], 188 [3], 198 [3, 4], 218 [7, 8], 240 [3, 4, 6], 294 [3, 4, 9], 362 [3, 4].

| Accession number  UniProt | Protein name | total number of tyrosine | Tyrosine nitration sites predicted | | Experimentally verified tyrosine nitration sites |
| --- | --- | --- | --- | --- | --- |
|  |  |  | GPS-YNO2 1.0 | iNitro-Tyr |  |
| Q9WVK5  O88829  Q64687  A0A1Z4EAV4  Q9Z0F0  Q11204  O35657  Q9JMH3  Q9JMH7  Q8BZL1  P60710 | β4GalT-VI  ST3Gal-V  ST8Sia-I  β4GalNAcT-I  β3GalT-IV  ST3Gal-II  NEU1  NEU2  NEU3  NEU4  Actb | 24  15  16  20  9  14  14  12  15  11  15 | 365-382  115-157  173  6-212  173  257  262-406  20  7-345  *  53-69-91-169-188-198-218-240-294-337-362 | 67-102-355-365-382  110-243-373  212-320-332  6-266-364  141-173-219-317  26-54-166-201-240-276  *  179-294-309-333  293-346  177  53-69-91-169-188-198-240-294-306-362 | n.d.  n.d.  n.d.  n.d.  n.d.  n.d.  n.d.  n.d.  n.d.  n.d.  53-69-91-169-188-198-218-240-294-362 |

**References**

1. Liu, Z., et al., GPS-YNO2: computational prediction of tyrosine nitration sites in proteins. Mol Biosyst, 2011. **7**(4): p. 1197-204.

2. Xu, Y., et al., iNitro-Tyr: prediction of nitrotyrosine sites in proteins with general pseudo amino acid composition. PLoS One, 2014. **9**(8): p. e105018.

3. Ghesquière, B., et al., In vitro and in vivo protein-bound tyrosine nitration characterized by diagonal chromatography. Mol Cell Proteomics, 2009. **8**(12): p. 2642-52.

4. Aslan, M., et al., Nitric oxide-dependent generation of reactive species in sickle cell disease. Actin tyrosine induces defective cytoskeletal polymerization. J Biol Chem, 2003. **278**(6): p. 4194-204.

5. Chantler, P.D. and W.B. Gratzer, Effects of specific chemical modification of actin. Eur J Biochem, 1975. **60**(1): p. 67-72.

6. Zhang, Q., et al., A method for selective enrichment and analysis of nitrotyrosine-containing peptides in complex proteome samples. J Proteome Res, 2007. **6**(6): p. 2257-68.

7. Sacksteder, C.A., et al., Endogenously nitrated proteins in mouse brain: links to neurodegenerative disease. Biochemistry, 2006. **45**(26): p. 8009-22.

8. Kanski, J., S.J. Hong, and C. Schöneich, Proteomic analysis of protein nitration in aging skeletal muscle and identification of nitrotyrosine-containing sequences in vivo by nanoelectrospray ionization tandem mass spectrometry. J Biol Chem, 2005. **280**(25): p. 24261-6.

9. Zhan, X. and D.M. Desiderio, The human pituitary nitroproteome: detection of nitrotyrosyl-proteins with two-dimensional Western blotting, and amino acid sequence determination with mass spectrometry. Biochem Biophys Res Commun, 2004. **325**(4): p. 1180-6.
