# Supplementary material for: Ganglioside Synthesis by Plasma Membrane-Associated Sialyltransferase in Macrophages
Source: Int J Mol Sci. 2020 Feb 5;21(3):1063. doi: 10.3390/ijms21031063 (PMC7043224; doi:10.3390/ijms21031063)
Supplement: Supplementary file 1 [file ijms-21-01063-s001.zip › Supplementary File/Supplementary File.pdf]

Figure S1

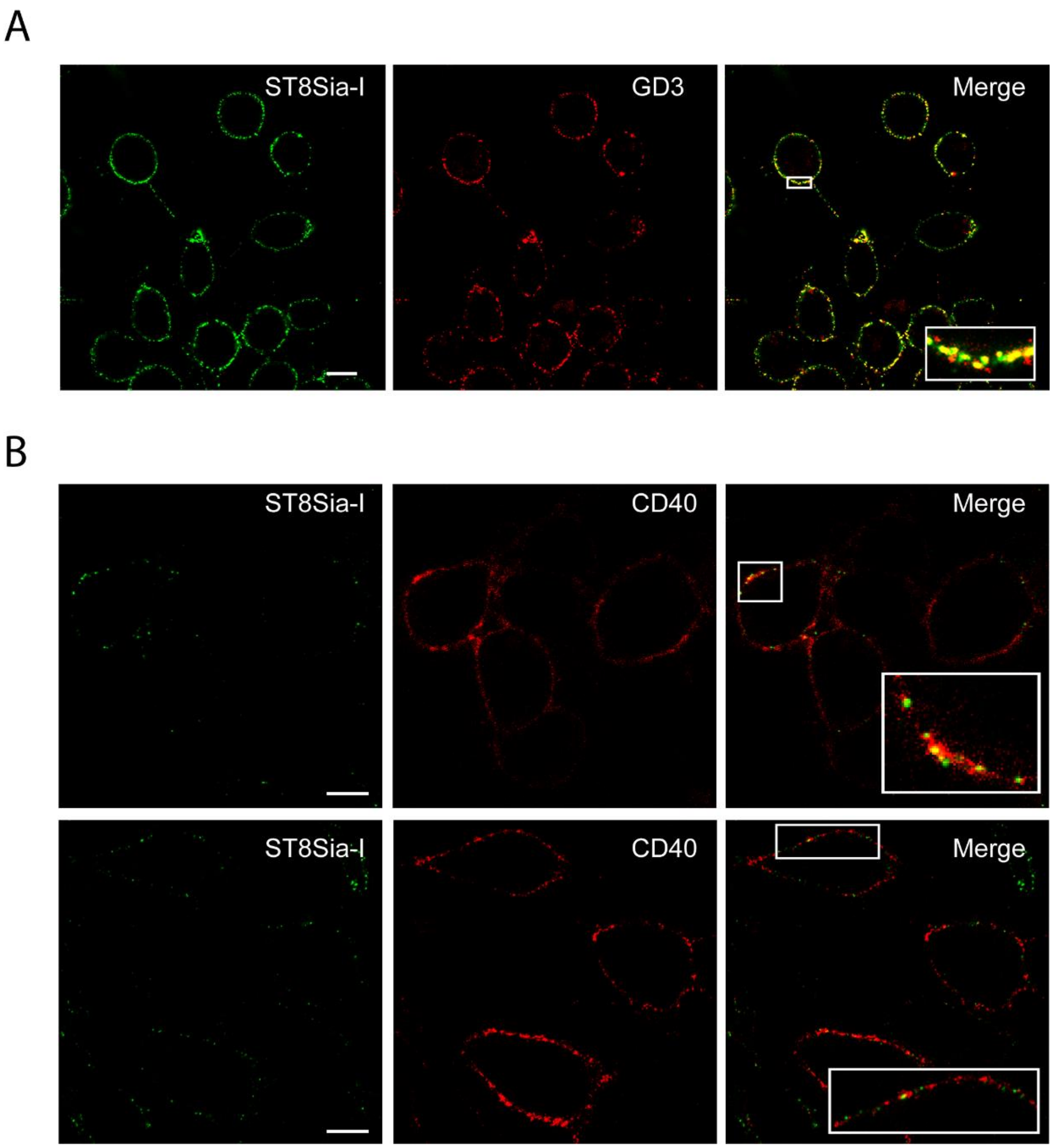

Immunofluorescence detection of ST8Sia-I, GD3 and CD40 at cell surface of RAW264.7 cell line. (**A**) Cells were immunostained with specific antibodies to ST8Sia-I and GD3 ganglioside at 4 °C for 60 min. Then, cells were fixed, incubated with secondary antibodies and visualized by confocal microscopy. Insets show details at higher magnification. (**B**) Cells were stimulated with LPS (LPS 100 ng/ml) for 48 h and immunostained with specific antibodies against ST8Sia-I and glycoprotein CD40 at 4 °C for 60 min. Then, cells were fixed, incubated with secondary antibodies and visualized by confocal microscopy. Insets show details at higher magnification. Scale bars:10 µm.

Figure S2

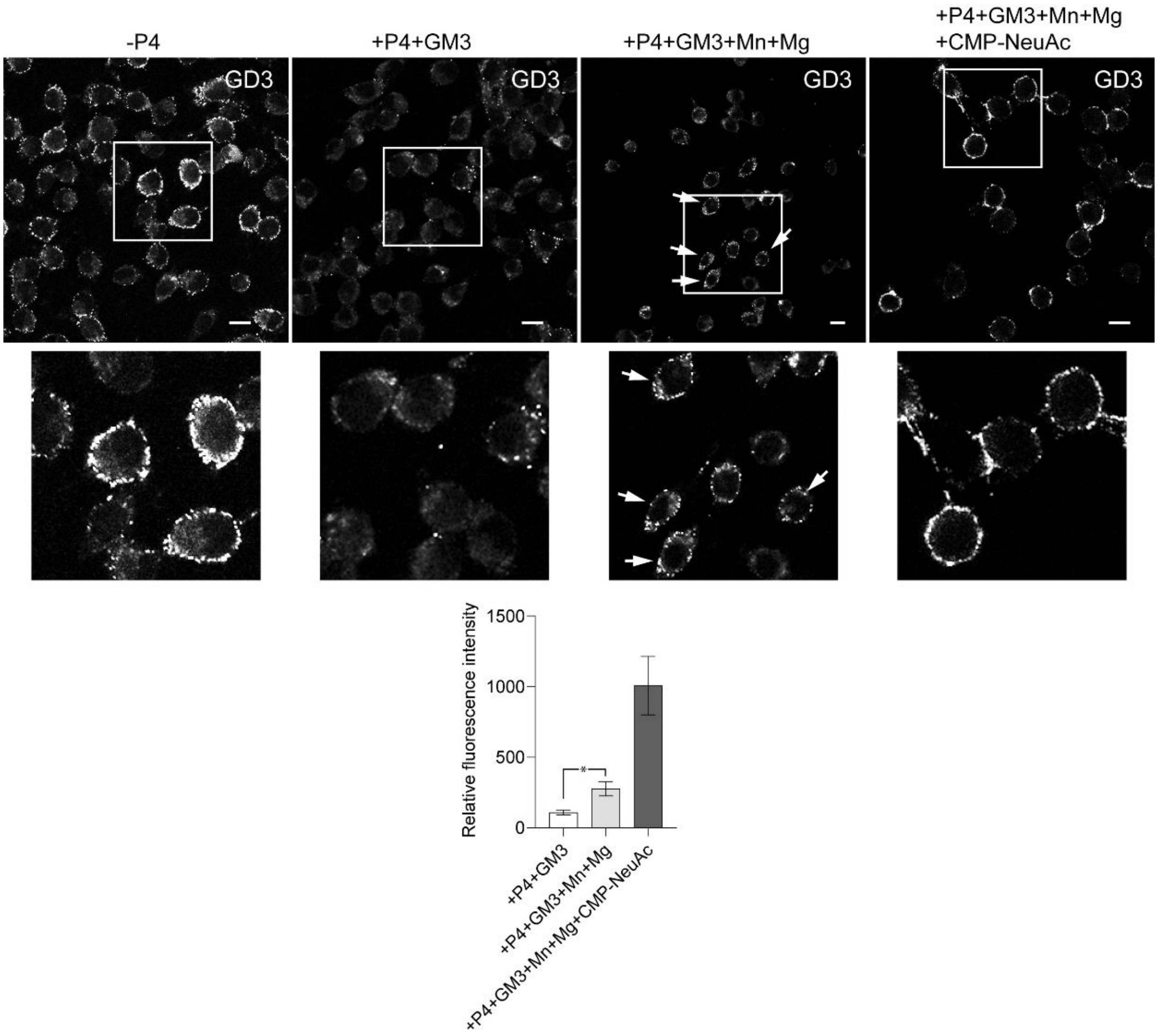

Ecto-ST8Sia-I sialylates exogenously incorporated GM3 in RAW264.7 cells. Macrophage RAW264.7 cells were grown with P4 or without P4 (−*P4*) for 4 days. Then, cells were treated with GM3, washed, and incubated at 37 °C for 1 h in a medium containing only DMEM (+*P4*+*GM3*) or in a medium containing Mn^{2+} and Mg^{2+} (+*P4*+*GM3*+*Mn*+*Mg*) or CMP-NeuAc, Mn^{2+} and Mg^{2+} (+*P4*+*GM3*+*Mn*+*Mg*+*CMP-NeuAc*). P4 inhibitor remained present throughout the experiments. Cells were washed, immunostained with antibody to GD3 at 4 °C for 60 min, and then fixed and incubated with secondary antibody conjugated to Alexa Fluor 488. Rows indicate GD3 synthesis. Insets show details at higher magnification. Single confocal sections were taken every 0.7 µm parallel to the coverslip. Scale bars:10 µm.

Table S1. Nitration sites in the listed proteins from mouse were computationally predicted using GSP-YNO2 1.0 and iNitro-Tyr software. "*" non-predicted site. "n.d." not determined. P60710 (actin, cytoplasmic 1), which nitrated Tyrosine residues have been identified experimentally (see references below) was using as cross-validation (please, see references [1, 2] for further information). ~ 95 % of the total number of tyrosine nitration sites were successfully indicated by the algorithms. 53 [3, 4], 69 [5], 91 [6], 169 [6], 188 [3], 198 [3, 4], 218 [7, 8], 240 [3, 4, 6], 294 [3, 4, 9], 362 [3, 4].

Accession number UniProt	Protein name	total number of tyrosine	Tyrosine nitration sites predicted		Experimentally verified tyrosine nitration sites
			GPS-YNO2 1.0	iNitro-Tyr	
Q9WVK5	β4GalT-VI	24	365-382	67-102-355-365-382	n.d.
O88829	ST3Gal-V	15	115-157	110-243-373	n.d.
Q64687	ST8Sia-I	16	173	212-320-332	n.d.
A0A1Z4EAV4	β4GalNAcT-I	20	6-212	6-266-364	n.d.
Q9Z0F0	β3GalT-IV	9	173	141-173-219-317	n.d.
Q11204	ST3Gal-II	14	257	26-54-166-201-240-276	n.d.
O35657	NEU1	14	262-406	*	n.d.
Q9JMH3	NEU2	12	20	179-294-309-333	n.d.
Q9JMH7	NEU3	15	7-345	293-346	n.d.
Q8BZL1	NEU4	11	*	177	n.d.
P60710	Actb	15	53-69-91-169-188-198-218-240-294-337-362	53-69-91-169-188-198-240-294-306-362	53-69-91-169-188-198-218-240-294-362

References

1. Liu, Z., et al., GPS-YNO2: computational prediction of tyrosine nitration sites in proteins. Mol Biosyst, 2011. **7**(4): p. 1197-204.
2. Xu, Y., et al., iNitro-Tyr: prediction of nitrotyrosine sites in proteins with general pseudo amino acid composition. PLoS One, 2014. **9**(8): p. e105018.
3. Ghesquière, B., et al., In vitro and in vivo protein-bound tyrosine nitration characterized by diagonal chromatography. Mol Cell Proteomics, 2009. **8**(12): p. 2642-52.
4. Aslan, M., et al., Nitric oxide-dependent generation of reactive species in sickle cell disease. Actin tyrosine induces defective cytoskeletal polymerization. J Biol Chem, 2003. **278**(6): p. 4194-204.
5. Chantler, P.D. and W.B. Gratzer, Effects of specific chemical modification of actin. Eur J Biochem, 1975. **60**(1): p. 67-72.
6. Zhang, Q., et al., A method for selective enrichment and analysis of nitrotyrosine-containing peptides in complex proteome samples. J Proteome Res, 2007. **6**(6): p. 2257-68.
7. Sacksteder, C.A., et al., Endogenously nitrated proteins in mouse brain: links to neurodegenerative disease. Biochemistry, 2006. **45**(26): p. 8009-22.
8. Kanski, J., S.J. Hong, and C. Schöneich, Proteomic analysis of protein nitration in aging skeletal muscle and identification of nitrotyrosine-containing sequences in vivo by

nanoelectrospray ionization tandem mass spectrometry. J Biol Chem, 2005. **280**(25): p. 24261-6.

9. Zhan, X. and D.M. Desiderio, The human pituitary nitroproteome: detection of nitrotyrosyl-proteins with two-dimensional Western blotting, and amino acid sequence determination with mass spectrometry. Biochem Biophys Res Commun, 2004. **325**(4): p. 1180-6.
