# Supplementary figures and images for: Ganglioside Synthesis by Plasma Membrane-Associated Sialyltransferase in Macrophages
Source: Int J Mol Sci. 2020 Feb 5;21(3):1063. doi: 10.3390/ijms21031063 (PMC7043224; doi:10.3390/ijms21031063)

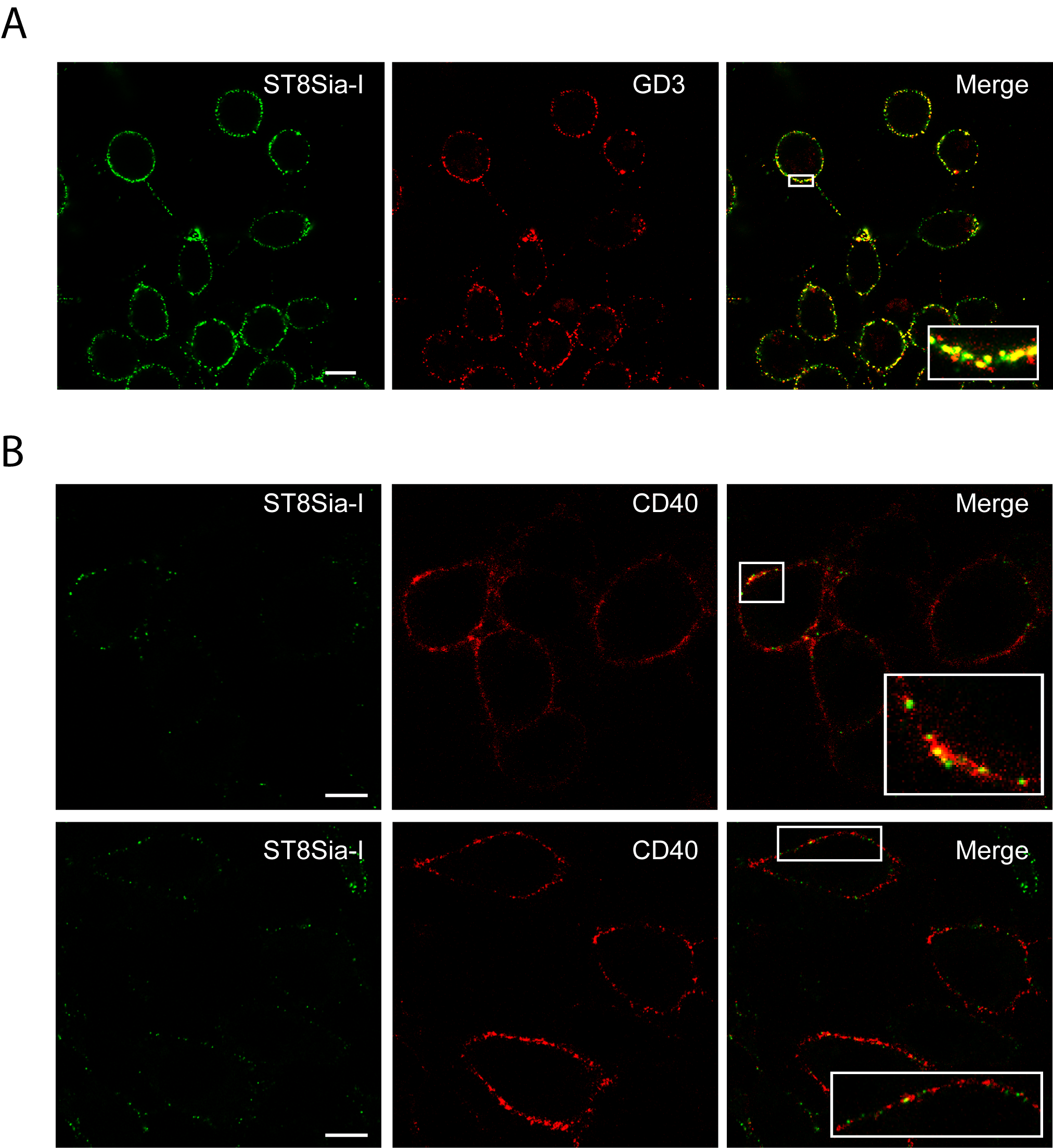

Supplement: Supplementary file 1 [file ijms-21-01063-s001.zip › Supplementary File/Figure S1.tif]

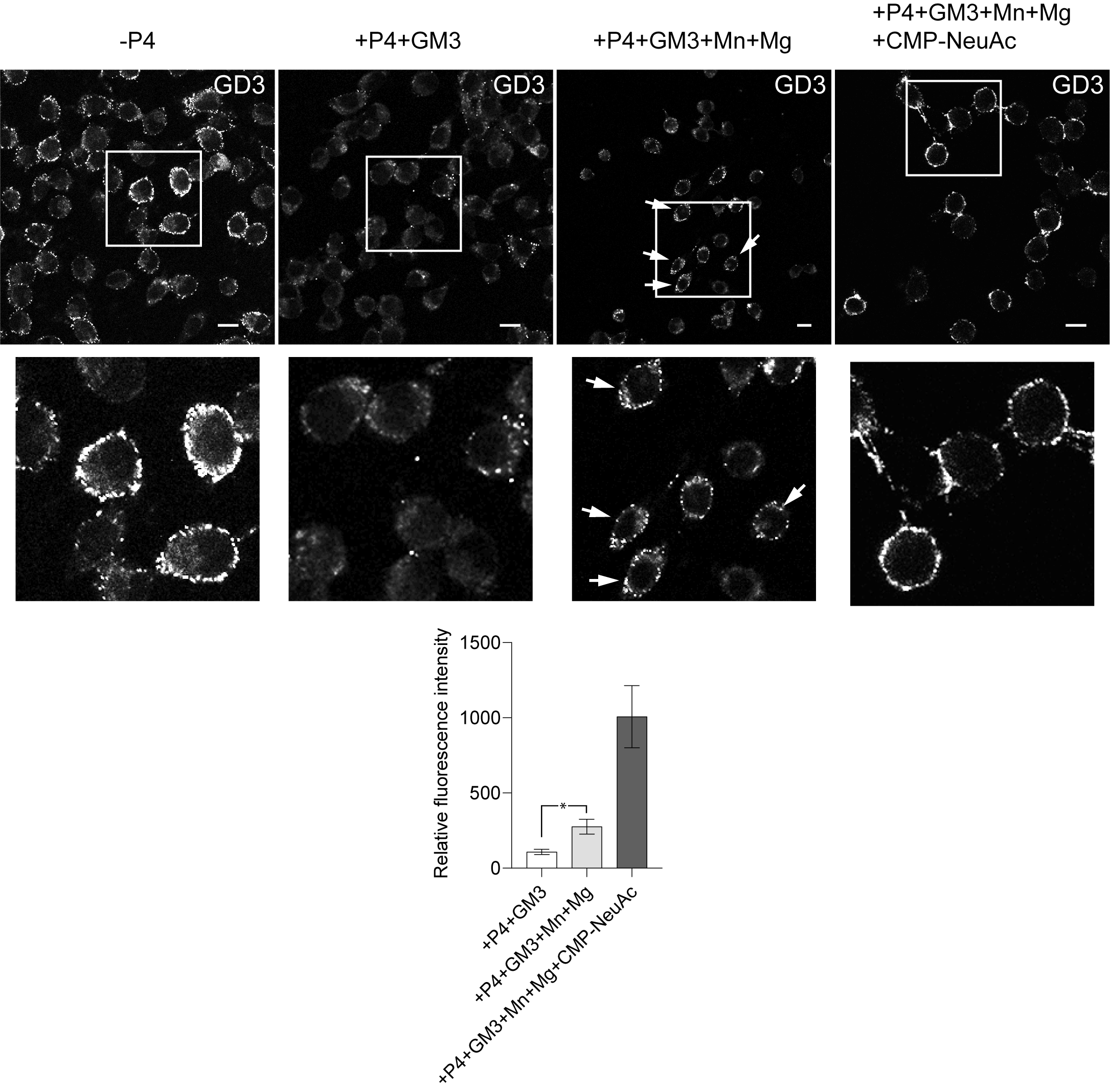

Supplement: Supplementary file 1 [file ijms-21-01063-s001.zip › Supplementary File/Figure S2.tif]
